# Music meets robotics: a prospective randomized study on motivation during robot aided therapy

**DOI:** 10.1186/s12984-018-0413-8

**Published:** 2018-08-16

**Authors:** Kilian Baur, Florina Speth, Aniket Nagle, Robert Riener, Verena Klamroth-Marganska

**Affiliations:** 1 Sensory-Motor Systems Lab, Department of Health Sciences and Technology, Swiss Federal Institute of Technology (ETH Zurich), Tannenstrasse 1, Zurich, 8092 Switzerland; 2 Spinal Cord Injury Center, University Hospital Balgrist, University of Zurich, Forchstrasse 340, Zurich, 8008 Switzerland; 3 Institute for Rehabilitation Science, Humboldt-Universitaet zu Berlin, Berlin, Germany, Unter den Linden 6, Berlin, 10099 Germany; 4 Institute of Occupational Therapy, School of Health Professions, Zurich University of Applied Sciences, Technikumstrasse 81, Winterthur, 8401 Switzerland

**Keywords:** Robotic arm rehabilitation, Creativity, Intrinsic motivation, Audio-haptic display, Music therapy, Serious games, Stroke, User interface

## Abstract

**Background:**

Robots have been successfully applied in motor training during neurorehabilitation. As music is known to improve motor function and motivation in neurorehabilitation training, we aimed at integrating music creation into robotic-assisted motor therapy. We developed a virtual game-like environment with music for the arm therapy robot ARMin, containing four different motion training conditions: a condition promoting creativity (C+) and one not promoting creativity (C–), each in a condition with (V+) and without (V–) a visual display (i.e., a monitor). The visual display was presenting the game workspace but not contributing to the creative process itself. In all four conditions the therapy robot haptically displayed the game workspace. Our aim was to asses the effects of creativity and visual display on motivation.

**Methods:**

In a prospective randomized single-center study, healthy participants were randomly assigned to play two of the four training conditions, either with (V+) or without visual display (V–). In the third round, the participants played a repetition of the preferred condition of the two first rounds, this time with a new V condition (i.e., with or without visual display). For each of the three rounds, motivation was measured with the Intrinsic Motivation Inventory (IMI) in the subscales interest/enjoyment, perceived choice, value/usefulness, and man-machine-relation. We recorded the actual training time, the time of free movement, and the velocity profile and administered a questionnaire to measure perceived training time and perceived effort. All measures were analysed using linear mixed models. Furthermore, we asked if the participants would like to receive the created music piece.

**Results:**

Sixteen healthy subjects (ten males, six females, mean age: 27.2 years, standard deviation: 4.1 years) with no known motor or cognitive deficit participated. Promotion of creativity (i.e., C+ instead of C–) significantly increased the IMI-item interest/enjoyment (*p*=0.001) and the IMI-item perceived choice (*p*=0.010). We found no significant effects in the IMI-items man-machine relation and value/usefulness. Conditions promoting creativity (with or without visual display) were preferred compared to the ones not promoting creativity. An interaction effect of promotion of creativity and omission of visual display was present for training time (*p*=0.013) and training intensity (*p*<0.001). No differences in relative perceived training time, perceived effort, and perceived value among the four training conditions were found.

**Conclusions:**

Promoting creativity in a visuo-audio-haptic or audio-haptic environment increases motivation in robot-assisted therapy. We demonstrated the feasibility of performing an audio-haptic music creation task and recommend to try the system on patients with neuromuscular disorders.

**Trial registration:**

ClinicalTrials.gov, NCT02720341. Registered 25 March 2016, https://clinicaltrials.gov/ct2/show/NCT02720341

## Background

Following a stroke, 80-90% of patients suffer from arm paresis, which remains chronic in about 30-40% of all cases [1–3]. Task-oriented, intensive, and motivational training is important to increase arm function post-stroke [2, 4–8].

Intensity is recognized as a key feature of successful rehabilitation therapy [9]. Robots in neurorehabilitation allow for highly-intensive, task-oriented training and have the potential to be superior to conventional therapies (i.e., physical or occupational therapy) in improving motor function post-stroke [10]. Robotic therapy may embed functional training tasks into computer games to facilitate motor learning and to stimulate motivation [11].

Autonomy, competence, and relatedness can be regarded as the main components of intrinsic motivation [12, 13]. While extrinsic motivation can be described as a goal-directed drive towards an externally provided reward (e.g., a score in a game), intrinsic motivation is a process oriented and internally provided reward due to a satisfying, interesting, meaningful or enjoyable activity [14, 15]. The knowledge regarding the meaningfulness of an activity is a positive determinant of patient motivation [7]. Thus, for patients, an activity should not only be enjoyable, but also lead to a rehabilitation progress. Furthermore, patient engagement is related to the expected reduction of impairment during game-based therapy in stroke [16].

Activities with a close relation to intrinsic motivation are frequently associated with activities promoting creativity [17–19]. This might be because activities promoting creativity involve one’s own accord, active decision making, and a resulting product, thus satisfying the need of autonomy, competence, and relatedness [12, 20–22].

In addition to encouraging creativity, music is a promising stimulator for intrinsic motivation in the context of rehabilitation [23, 24]. Music effectively promotes post-stroke recovery in motor and cognitive functions, and furthermore in emotional and social domains [25–31]. Studies that compared conventional therapy forms to therapy tasks embedded in active music making revealed that music-associated training increases the level of motivation significantly [24, 32].

Auditory displays have already been determined to be effective for navigation within complex systems [33]. Accordingly, sound is an audible source for navigation through the execution of a task in virtual scenarios without the need for a visual display unit, the advantage being that the visual focus can be on the trained limb rather than a graphical display, thus promoting visuo-motor control [34, 35].

We developed tasks for robot-assisted training of the arm that aim to increase intrinsic motivation with a focussed stimulation of the two aspects: creativity and music. To investigate whether a music condition promoting creativity influences motivation differently than a music condition not promoting creativity, we compared motivational effects of both versions. We investigated the effect of the presence or absence of a visual display for both conditions regarding promotion of creativity. As the training goal of the presented gamified task is to induce high intensity during exercise, the game is operated by repetitive horizontal movements.

For this current study, we designed audio-haptic tasks in a way that they can be performed either with visual display (i.e., a monitor presenting the game workspace) as an audio-visuo-haptic environment or without a visual display as an audio-haptic environment only. To reduce the cognitive load of the participants and have more cognitive resources for creation and decision making processes, we designed the visual display and the haptic environment such that they both presented the same game workspace [36]. Accordingly, the visuals were not essential to complete the audio-haptic task.

Given these related works, the primary hypothesis was that a gamified task promoting creativity embedded in a task for motor therapy increases intrinsic motivation more than a gamified task not promoting creativity. Our second hypothesis was that a gamified task in motor therapy without visual display increases intrinsic motivation more than a gamified task with visual display. Moreover, we hypothesized that promoting creativity and omitting a visual display would increase total training time, free movement time and perceived product value. We further hypothesized that promoting creativity and omitting a visual display would reduce energy expenditure, relative perceived training time and perceived effort.

## Methods

We conducted a prospective randomized single-center study in Zurich, Switzerland. Approval was obtained from the responsible Ethical Committee (KEK-ZH-Nr. 2015-0013, Zurich, Switzerland). The study is registered at ClinicalTrials.gov (identifier: NCT02720341).

### Subjects

Sixteen healthy subjects with no known motor or cognitive deficit were to be recruited among the population of Zurich. Most of them were employees and students of ETH Zurich.

### Technical setup

**Fig. 1:**
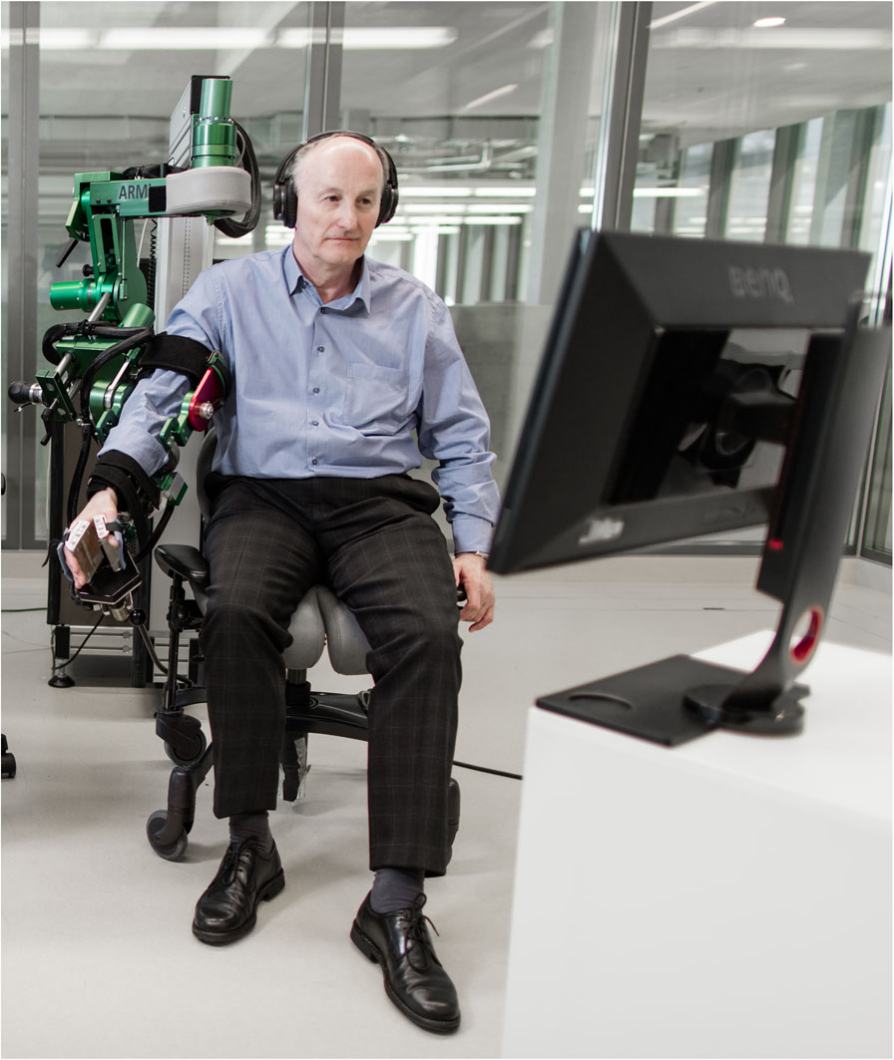
ARMin arm rehabilitation robot. Additionally for this study, a keyboard was placed close to the participant’s left hand so that the space bar could be used as input device

The study was performed with the ARMin arm rehabilitation robot generation IV shown in Fig. 1. ARMin is a seven-degree-of-freedom exoskeleton robot for arm therapy of patients with neurological disease. It was developed by the groups of Riener at ETH Zurich and Dietz/Curt at the University of Zürich [37, 38] (technical details: see Appendix ARMin).

### Game environment

The game environment was developed targeting horizontal movements at table height as this type of arm motion is required for activities of daily living, such as cleaning a table or moving objects on a table. Such training activities are already provided in several upper limb training devices for stroke therapy [38, 39].

Four conditions were developed in the Unity game engine [40]: A music task promoting creativity played without a visual display (C+V–), a music task promoting creativity played with a visual display (C+V+), a music task not promoting creativity played without a visual display (C–V–) and a music task not promoting creativity played with a visual display (C–V+). All four conditions used the same audio-haptic environment. In this environment, participants have to select one out of two sound samples positioned to the left and to the right, respectively, several times per condition. In the conditions with visual displays, the environment was complemented with visual feedback (audio-visuo-haptic environment).

**Fig. 2:**
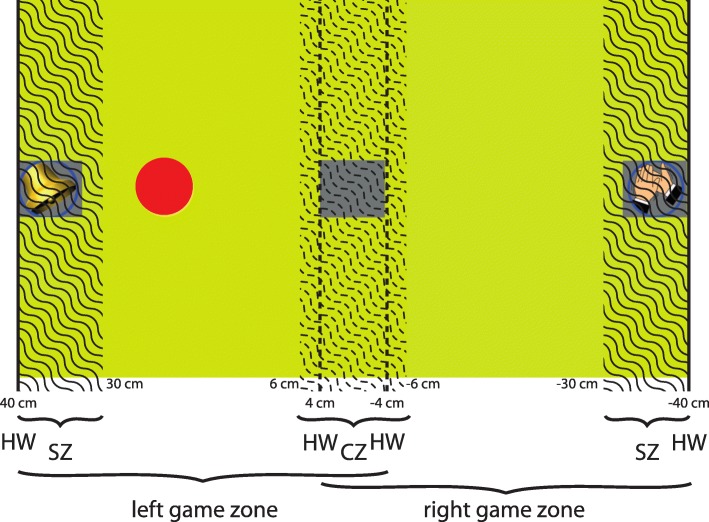
Screenshot of the tasks in V+ conditions with additional map of haptic elements: The visual environment displays sound icons (e.g., a bell or hands clapping) within sound zones (SZ), and a grey square that marks the center zone (CZ). SZs and CZ (wave-marked areas) are active zones where a vibrotactile haptic feedback is provided. The scene is limited by two haptic walls (HW, solid lines). HWs (dotted lines) limiting CZ are only turned on in the game phases where only the left or the right game zone is used. The red circle is the game “cursor”. All units are in centimeters

To provide task oriented training we haptically simulated an environment of moving objects on a table. Subjects could only move the end effector of the robot horizontally. Downward movements were restricted by a virtual table that was set at the level of the shoulder and provided arm support. The horizontal left-right movement served as game input. Therefore, the game can be considered as a one-degree-of-freedom task. As illustrated in Fig. 2, the haptic display included haptic walls at the end of the workspace (HWs). Sound zones (SZ) and center zones (CZ) were action zones where a vibrotactile haptic feedback (i.e., band-limited white noise) was provided at the hand module of the robot. The vibrotactile haptic feedback was induced via DC motors at the wrist joint of the robot. As a perceivable state indicator (i.e., being inside or outside of a SZ) for the participants, the vibrotactile haptic feedback was not breaking or stopping the participant’s movement. None of the participants commented on the vibrotactile feedback.

While in C–V+ and C+V+, the task was visualized on a monitor, in C–V– and C+V–, the same movements were performed without visual feedback (i.e., without a monitor).

The sounds used in the SZs consisted of fourteen different pairs of sound samples and two pairs of sound effects. These sixteen pairs of music creation elements were presented to the participant in fixed order. The sound samples consisted of synthetic piano, mallets, marimba, vibraphone, pads, drums, hi-hats, and claps. Harmonic elements were tuned in C-Major. Each sound sample lasted six seconds and was played in a loop with a tempo of 80 beats per minute. The two pairs of sound effects (i.e., modulators) were Reverb and Echo, and Resonance and Arpeggiator. To ensure a well-formed, aesthetic and pleasant music structure, the sound samples and the modulators were designed such that they suit to each other when playing simultaneously (see Appendix Sound examples). The underlying music composition was developed with the commercial music software Abelton Live 8 (Abelton). Subjects listened to the sounds using Sony MDR-7506Ⓡ headphones.

All conditions used the same audio-haptic environment wherein the haptic walls together with instrument sounds gave feedback about the position in the game.

### Game rules

**Fig. 3:**
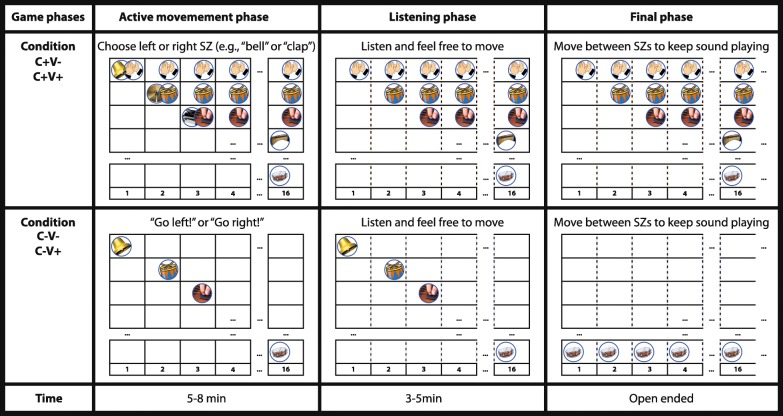
Game rules for all four conditions. C+ conditions and C– conditions for all three game phases (i.e., active movement phase, listening phase, final phase) are presented. In C+ conditions, four successively activated sounds add up to a music piece (upper field, from top to bottom: claps, drums, guitars, marimba, …). In C– conditions (lower field), each consecutively activated sound is played alone

The game rules for all four conditions are illustrated in Fig. 3. Each of the four conditions consisted of of three game phases in fixed order, namely an active movement phase, a listening phase, and a final phase). The three game phases were different in the conditions promoting creativity (C+) and in the conditions not promoting creativity (C–) but independent of the inclusion or omission of a visual display.

The only difference between the V+ and the V– conditions was the workspace visualization on a monitor which was either provided (V+) or omitted (V–).

#### Detailed game rules for the C+ condition

In the active movement phase of C+ conditions, two sounds were presented, one in the right and one in the left SZ. By moving the arm (fixed in ARMin) into the SZs, the subject could listen to the corresponding sound. Pressing the spacebar key while being in the SZ activated that sound. Activating a sound caused that sound sample to play in a loop. Once a sound was activated, subjects had to move between CZ and SZ. The more they moved, the more the volume increased. The range of volume was from no sound to comfortable volume, as assessed during study instruction. When they stopped moving, the sound was played in a loop with low volume. After at least three repetitions, the subject could start the next round by pressing the space key.

In the listening phase, the resulting music composition was played back to the subjects at a constant volume. Subjects were invited to listen only or to move to the music freely.

In the final phase, the last four activated sounds of the piece were played back. To keep the volume up, movement between the two SZs was needed. As soon as no movement reaching out to the SZs was performed, the volume decreased. The final phase ended whenever the user wanted.

#### Detailed game rules for the C– condition

In the active movement phase of the C– conditions, subjects could not choose sounds. They followed verbal instructions, either “go left” or “go right”. Upon moving to the correct SZ, the related sound was played. After five movements between SZ and CZ, the next instruction was given. Two additional movements had to be made in the C- condition compared to the three movements in the C+ condition to balance out the expected exploring behavior of the participants in the C+ condition. This exploring behavior was assumed to be at least one movement towards each side. The direction was randomly generated for each round. This was performed over fourteen sounds and two sound effects. Although participants never tried to cheat, i.e., go into the opposite direction than instructed, would not have been possible. The next round of sounds are not played unless instructions are followed.

In the listening phase, the resulting music composition was played back to the subjects at a constant volume. Subjects were invited to listen only or to move to the music freely.

In the final phase, the presented sound consisted of the last four sounds activated in the active movement phase. These sounds were displayed serially and in a loop. To keep the volume up, subjects had to continue the movements between the two SZs. As soon as no movement was performed, the volume decreased. The final phase ended whenever the user wanted.

### Study procedure

Considering the exploratory type of the study and to counterbalance the groups, we decided that *n*=16 was a possible subject count [41]. Accordingly, we created sixteen cards with the four groups A, B, C, D printed on it. Table 1 shows the four groups and their corresponding conditions. Each new subject was asked to draw from this deck of cards. Subjects were assigned the group and an increasing ID within the group, according to the card they drew. A card once drawn was discarded. In the end we achieved random assignment for the sixteen subjects among the four groups.

**Table 1:** Groups A, B, C, D (4 subjects each): order of conditions for rounds 1-3; C+= promoting creativity; C–= not promoting creativity; V+ = with visual display; V– = without visual display

Round	1	2	3
Group A	C+V+	C–V+	Preferred task without visual display (C+V– or C–V–)
Group B	C–V+	C+V+	
Group C	C+V–	C–V–	Preferred task with visual display (C+V+ or C–V+)
Group D	C–V–	C+V–	

The subject was seated in ARMin with their right arm fixed to the device. An audio guide explained the game rules with the example of C+V+. It explained how to position and move the arm to first explore and then activate sound samples and sound effects. Two repetitions of C+V+ were explained through verbally instructed game playing by the audio guide which was triggered by game states. The audio guide explained also, that in the listening phase the composed music was replayed, and that in the final phase a movement stop would decrease the sound volume until the participant wanted to stop the round by telling the experimenter. Finally, the differences to other conditions, i.e., being instructed where to move (C–) and playing without a visual display (V–), were explained by the audio guide.

After each round, the subject filled in the questionnaires. After the second round, the subject selected a preferred condition. According to that selection, the third round was performed in the preferred creativity condition, i.e., C+ or C–, with the not played visual display condition, i.e., V+ or V–. In order to be able to use the same study protocol for patients at a later time, we aimed to minimize the study time and implicitly the strain for the individual participant. Thus, each subject performed only three of the four possible conditions.

Comments stated by the participants during or after each round were transcribed by the examiner.

### Outcome measures

#### Primary outcomes

The Intrinsic Motivation Inventory (IMI) is a multidimensional measurement device for assessment of participants’ subjective experience related to a specific activity. While there are many versions of the IMI, in this study, statements of the subscales interest/enjoyment (seven statements), perceived choice (seven statements), value/usefulness (two statements), man-machine-relation (five statements, in IMI original name of item: relatedness) were used [42]. The statement sentences for each sub-item were presented and subjects answered on a Likert scale ranging from 1 to 7 (1: do not agree at all; 7: fully agree).

From the subscale interest/enjoyment and perceived choice all available statements were used. Interest/enjoyment is the only subscale that is an indicator of the subjective experience per se while the other subscales are related to the satisfaction of basic psychological needs that promote intrinsic motivation [14]. The perceived choice is related to the psychological need for autonomy and competence.

The subscale value/usefulness was represented by the statements “I believe composing music could be of some value to me.” and “I would be willing to do this again.”. The value/usefulness subscale measures in how far people internalize and become self-regulating with respect to activities that they experience as useful or valuable for themselves.

Finally, the subscale man-machine relation was represented by the statements “I felt really distant to the robot.”, “I don’t feel like I could really trust the robot.”, “I’d like a chance to interact with the robot more often.”, “I’d really prefer not to interact with the robot in the future.”, and “I felt like I could really trust the robot.”. Man-machine-relation evaluates the degree of a person’s feelings towards and interactions with the device.

#### Secondary outcomes (recorded)

During each task performance, the ARMin system recorded the total training time, time of free movement, i.e., duration of final phase, and root-mean-square (RMS) of the end effector velocity profile. RMS of the end effector velocity profile approximates the energy expenditure [43] and was recently used in neuromuscular therapy studies [44].

#### Secondary outcomes (self-reported)

After the performance of the first two tasks, the subject was asked to report the most preferred task. The subject was instructed to estimate the perceived training time after each task. This perceived training time was compared later to the actual training time (relative perceived training time). Additionally, the perceived effort was rated: Via ratings along a Borg Scale from one to twenty the subject grades, how exhausting the task is perceived [45]. After each task, the subject was asked whether he or she would like to receive the created music piece as mp3. Their answer was used to assess the perceived product value.

#### Subject data

We assessed age and sex of each subject. Furthermore, personality profiles were screened with the Ten Item Personality Inventory (TIPI). In the TIPI test, statement sentences are presented that are related to ten personality traits. Test subjects are instructed to assign to which degree they identify with these traits along a Likert scale ranging from 1 to 7 (1: do not agree at all; 7: fully agree). Creativity was evaluated with the sub-test on verbal creativity out of the Torrance Test of Creative Thinking (TTCT) [46]. This test measures verbal creativity along the two dimensions fluency (number of words) and flexibility (number of different semantic categories of relevant responses). A stimulus word (in our case “garden”) is presented. The task is to list as many words as possible associated with the stimulus word within 60 seconds. TIPI and TTCT were assessed to get an overall impression of the study population’s variability regarding personality and creativity.

### Statistical analysis

We used linear mixed models to analyze each IMI-item (interest/enjoyment, perceived choice, value/usefulness, man-machine relation), the recorded measures (training time, free movement time, RMS of the end effector velocity profile), and the self-reported measures relative perceived training time and perceived size of effort. The only random effect was the individual subject. We determined significant effects of promoting creativity (C+ instead of C–), the omission of a visual display (V– instead of V+), and interaction effects. The significant effects were presented as within-subject corrected (i.e., relative to the mean value of the subject over all performed tasks).

For the preferred task, we reported the number of subjects who stated a task as being their preference. For the perceived size of product value, we reported the number of positive and negative answers.

## Results

Sixteen healthy subjects (ten males, six females, mean age: 27.2 years, standard deviation: 4.1 years) with no known motor or cognitive deficit participated in this study. The detailed results are reported in the Appendix Summary of results in Table 5. We visually inspected the residuals of the linear mixed models.

### Primary outcomes

**Fig. 4:**
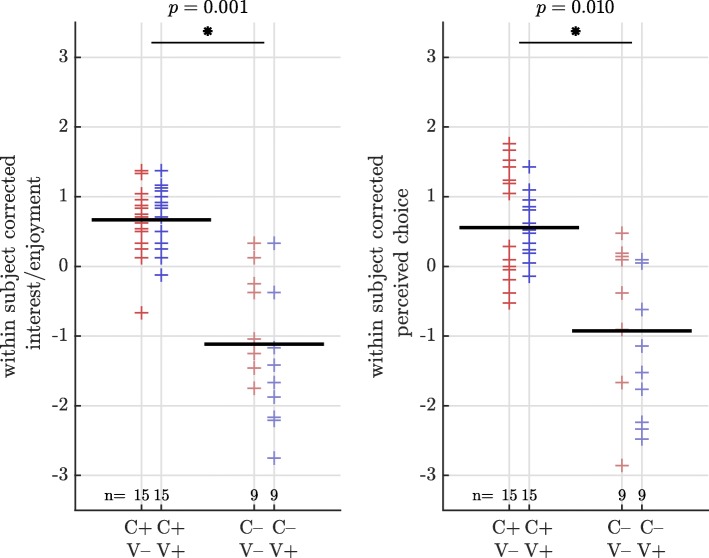
Within subject corrected rating of subscale interest/enjoyment (left) and perceived competence (right) for visualization of the significant effect in promotion of creativity (C+ instead of C–) using linear mixed models. The black line indicates the mean of the performances the line is crossing. Each condition was played by a different number of subjects (n)

Linear mixed models analysis of the IMI subscales interest/enjoyment and perceived choice showed a significant positive effect of promoting creativity (C+ instead of C–) as presented in Table 2 and Fig. 4. No significant effects for man-machine relation and value/usefulness were found.

**Table 2:** Linear mixed models analysis of the fixed effects promotion of creativity and omission of visual display on the IMI subscales interest/enjoyment, perceived competence, man-machine relation and value/usefulness

	Mean (std. error)	t	p	95% CI	
				Lower	Upper
Interest/enjoyment					
Intercept	5.6(0.2)	26.5	0.000	5.1	6.0
No creativity	− 1.2(0.4)	− 3.4	0.001	− 1.9	− 0.5
Visual display	0.1(0.1)	0.4	0.666	− 0.3	− 0.4
Interaction	− 1.1(0.6)	− 1.9	0.061	− 1.1	0.1
Perceived choice					
Intercept	4.7(0.3)	14.6	0.000	4.1	5.4
No creativity	− 1.2(0.4)	− 2.7	0.010	− 2.1	− 0.3
Visual display	0.0(0.4)	0.0	0.980	− 0.8	− 0.8
Interaction	− 0.9(0.5)	− 1.6	0.107	− 0.9	0.2
Man-machine relation					
Intercept	5.3(0.3)	18.6	0.000	4.7	5.8
No creativity	0.0(0.2)	0.0	0.976	− 0.4	− 0.4
Visual display	0.0(0.2)	0.1	0.959	− 0.4	0.4
Interaction	0.0(0.4)	− 0.1	0.943	0.0	0.8
Value/usefulness					
Intercept	4.9(0.3)	15.3	0.000	4.2	5.5
No creativity	− 0.8(0.5)	− 1.7	0.101	− 1.8	0.2
Visual display	0.2(0.2)	1.2	0.252	− 0.1	0.5
Interaction	− 0.9(0.7)	− 1.2	0.228	− 0.9	0.6

### Secondary outcomes (recorded)

Linear mixed models of the total training time and RMS of the velocity profile showed a significant interaction effect in promotion of creativity and omission of visual display as presented in Table 3. In the conditions without a visual display, promotion of creativity leads to less total training time and higher RMS of velocity while the opposite effects are found in conditions with a visual display. No significant effects in free movement time were found.

**Table 3:** Linear mixed models analysis of the fixed effects promotion of creativity and omission of visual display on total training time, free movement time and RMS of velocity

	Mean (std. error)	t	p	95% CI	
				Lower	Upper
Training time [*s*]					
Intercept	518(29)	17.6	0.000	458	577
No creativity	236(139)	1.7	0.096	−44	516
Visual display	38(40)	0.9	0.348	−42	118
Interaction	− 387(149)	− 2.6	0.013	− 387	− 87
Free movement time [*s*]					
Intercept	133(12)	10.8	0.000	108	157
No creativity	− 37(24)	− 1.5	0.135	− 86	12
Visual display	16(17)	0.9	0.965	− 19	51
Interaction	− 20(50)	− 0.4	0.688	− 20	81
RMS of the velocity profile					
Intercept	0.09(0.01)	9.20	0.000	0.07	0.11
No creativity	− 0.02(0.01)	− 1.70	0.092	− 0.05	0.00
Visual display	− 0.01(0.01)	− 0.50	0.606	− 0.04	0.02
Interaction	0.07(0.02)	3.80	0.000	0.07	0.11

### Secondary outcomes (self-reported)

**Fig. 5:**
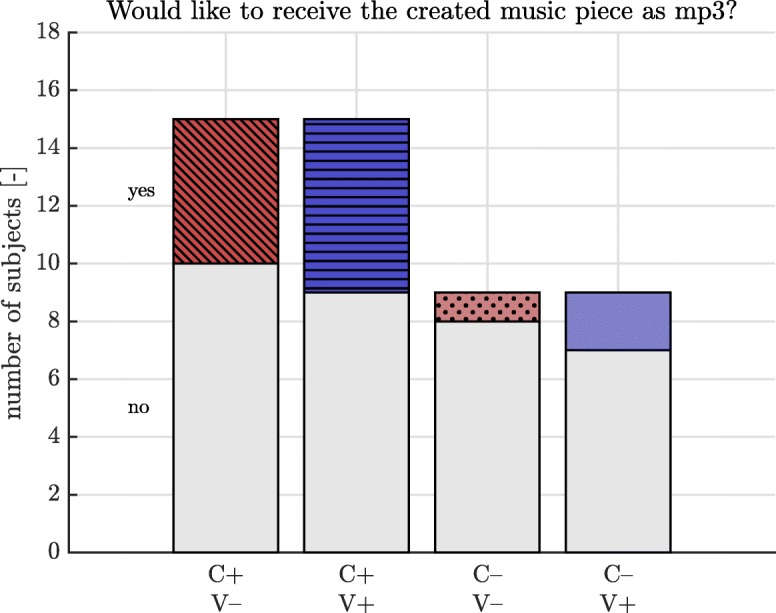
Perceived size of value indicated by the desire to receive the music on mp3

Fourteen out of sixteen subjects preferred the conditions promoting creativity (C+) to the conditions not promoting creativity. Linear mixed models of the relative perceived training time and of the perceived size of effort showed no significant effect in promotion of creativity and omission of visual display as presented in Table 4. In the C+ conditions, the subjects wanted to receive the created music as a file in eleven of 30 trials. In the C– conditions, as shown in Fig. 5, the subjects wanted to have the created music as a file in three out of eighteen trials.

**Table 4:** Linear mixed models analysis of the fixed effects promotion of creativity and omission of relative perceived training time and perceived size of effort

	Mean (std. error)	t	p	95% CI	
				Lower	Upper
Relative perceived training time [s]					
Intercept	74(50.4)	1.5	0.148	−27.3	175.3
No creativity	− 133.3(100.5)	− 1.3	0.191	− 335.2	68.7
Visual display	− 4.9(76.8)	− 0.1	0.949	− 159.3	149.4
Interaction	91.8(149)	0.6	0.540	91.8	391.0
Perceived size of effort [-]					
Intercept	5.1(0.8)	6.8	0.000	3.6	6.6
No creativity	1.1(1.2)	0.9	0.378	− 1.4	3.6
Visual display	0.4(0.7)	0.6	0.568	− 1.0	1.9
Interaction	− 1.2(2.5)	− 0.5	0.623	− 1.2	3.7

### Subject data

The TIPI stated a mean value of 4.31 in extraversion (standard deviation: 0.83), 4.56 in agreeableness (0.85), 4.44 in conscientiousness (0.54), 3.72 in emotional stability (1.02), and 4.09 in openness to experience (0.55). The TCTT stated a mean value of 5.06 in flexibility (standard deviation: 1.00) and 14.94 in fluency (3.64).

## Discussion

We could prove that intrinsic motivation is increased by tasks promoting creativity when performing a robot-assisted music task. The tasks promoting creativity were preferred and rated more enjoyable than those without creativity. These results are consistent with previous findings [47] and support the use of music to engage in robot-assisted training. In addition, the game modes that promoted creativity led to increased self-reported autonomy (indicated by IMI subscale of perceived choice).

Promoting creativity had no significant effect on usefulness. However, more subjects wanted to receive the music piece produced in modes where creativity was promoted. Therefore, process (i.e., creation of music piece) and product (i.e., music piece itself) must be discussed separately in game design regarding value.

The omission of a visual display did not affect intrinsic motivation ratings. According to statements of participants, playing without a visual display has been a new and exciting experience for them. However, without a visual display the rules might not be intuitively understood. Non-intuitive game rules may lower the feeling of competence [14]. Although not systematically measured, subjects reported verbally that the conditions without visual display were challenging. Therefore, future studies should measure the IMI subscale perceived competence. Furthermore, the conditions without visual display might benefit from a more detailed audio guide. Accompanying the users throughout the first steps of the game verbally may increase the feeling of competence. For patients in neurorehabilitation therapy it might be even more challenging to understand the task [48]. For these patients, intuitive game design and clear game instructions are especially important. However, playing without a visual display facilitates the use of vision for movement observation which has a positive impact on recovery of motor functions [49]. Furthermore, vision could focus on human-human interaction. Seeing each other is particularly motivating while playing with training devices [50]. In our setup, we expected that the subjects would visually observe the human-robot interaction resulting in a higher man-machine relation. From observation we realized that subjects mainly looked straight ahead to the place where usually a monitor would be expected. As stated by the subjects, they were rather focusing on the auditory display of the game than on the arm movement. Likewise, no difference in man-machine relatedness was found. In further studies without visual displays, patients should be instructed, to visually focus on the arm movements [34, 35].

It is a principal objective of robot-assisted therapy to increase the intensity (i.e., energy expenditure, duration of therapy, number of repetitions) during therapy. In our study, creativity promoting tasks increased energy expenditure without visual display. However, (as shown by significant interaction) energy expenditure decreased with visual display. The possibility to explore the audio-haptic setting seemed to promote more intense moving behavior. On the contrary, when the target position was predefined and visualized (C–V+) the subjects moved fast with fewer slow movement phases for decision making. The subjects tried to reach the stated target positions (i.e., “go left”, “go right”) as fast as possible without resting phases for listening. However, in the condition with visual display where creativity was promoted (C+V+) the movements slowed down. In summary, we assume that when there is no visualisation of a reaching task ((V–), i.e., no extrinsically motivators for fast reaching movements), tasks promoting creativity may intrinsically motivate for more intensive movements.

Regardless of the conditions, the subjects voluntarily played on average two additional minutes within each eight minutes task. Obviously, the subjects were motivated to voluntarily extend time of arm movement after finishing the guided phases.

Future work could provide haptic guidance or resistance [51]. The decision to use or omit a visual display could be to the user. Alternatively, therapy could start with visual display until proficiency increases and be omitted later, e.g.,as an adaptation in task difficulty.

**Table 5:** Summary of the results for all groups A, B, C, D (4 subjects each) and all conditions: C+= promoting creativity; C-= not promoting creativity; V+ = with visual display; V- = without visual display

	Interest/enjoyment				Perceived choice				Man-machine relation				Value/usefulness				Training time (TT)				Free movement time				RMS of velocity				Rel. perceived TT				Perceived size of effort			
Units	–				–				–				–				*s*				*s*				*m*/*s*				*s*				*s*			
Mean	4.7				3.9				5.2				4.3				557				117				.087				123				5.3			
SD	1.3				1.3				1.0				1.4				236				79				.040				277				3.9			
	C+V–	C+V+	C–V–	C–V+	C+V–	C+V+	C–V–	C–V+	C+V–	C+V+	C–V–	C–V+	C+V–	C+V+	C–V–	C–V+	C+V–	C+V+	C–V–	C–V+	C+V–	C+V+	C–V–	C–V+	C+V–	C+V+	C–V–	C–V+	C+V–	C+V+	C–V–	C–V+	C+V–	C+V+	C–V–	C–V+
Mean	5.6	5.7	4.2	3.4	4.7	4.7	3.5	2.6	5.3	5.3	5.1	5.3	4.9	5.1	3.9	3.3	521	557	752	398	134	150	101	82	.087	.079	.063	.127	55	115	-18	15	4.6	5.3	6.6	5.3
SD	.8	.7	1.0	1.1	1.2	.8	.8	.7	1.0	.9	1.0	1.0	1.3	1.1	.8	1.3	112	128	404	117	47	68	78	108	.035	.040	.030	.020	207	302	335	229	2.8	4.3	3.7	4.1
A1	5.5	5.5		1.4	5.9	4.6		1.9	5.5	5.5		5.5	5.0	5.0		2.5	519	460		337	119	133		28	.056	.084		.116	-219	20		-37	2	2		15
A2	6.4	6.8		3.8	4.6	5		3.9	5.5	5.5		5.3	7.0	7.0		5.5	534	655		500	152	260		62	.117	.132		.096	-54	-55		40	2	4		8
A3	5.9	5.4		2.4	3.7	4.3		2.3	5.3	6.5		3.8	6.0	5.5		1.5	486	462		460	131	119		27	.072	.098		.137	414	438		-160	9	8		2
A4	5.4	5.1		2.8	5.9	5.7		2.3	3.8	4.3		4.3	5.0	5.0		3.0	478	599		670	156	179		77	.110	.071		.109	122	1		-250	5	4		8
B1		5.5	5.4	4.9		3.9	3.1	3.6		4.5	5.0	5.3		4.0	4.0	4.0		307	690	322		34	31	382		.184	.072	.140		293	-90	578		15	10	4
B2	6.6	6.1		4.6	5.6	4.7		2.9	7.0	6.8		6.8	4.5	4.5		4.5	313	521		369	99	100		81	.103	.059		.145	47	-221		-129	2	2		4
B3	6.3	6.4		3.0	6.0	5.1		1.9	5.5	5.5		7.0	2.5	3.5		1.5	565	892		286	97	89		25	.042	.033		.160	-145	-412		-106	1	1		1
B4	5.3	6.0		3.5	5.4	4.1		2.1	4.3	5.5		5.3	5.5	6.5		3.0	509	646		316	106	96		29	.083	.084		.134	91	-46		104	9	5		3
C1	4.1	4.5	2.1		3.4	4.0	4.4		4.5	5.0	5.5		3.5	3.5	3.0		688	540	521		226	139	63		.036	.037	.047		-88	60	-101		1	1	3	
C2	5.3	6.0	3.8		6.4	6.0	3.7		6.8	7.0	7.0		4.0	5.0	3.0		507	438	536		105	96	35		.115	.071	.058		-267	-198	-116		5	8	7	
C3	6.6	6.6	4.0		6.1	6.1	1.9		7.0	5.5	6.0		7.0	7.0	3.5		682	615	655		46	123	115		.024	.027	.023		-82	285	245		2	2	5	
C4	3.6		4.6	4.6	2.7		3.0	3.0	4.0		4.3	4.5	3.0		4.0	4.0	503		328	325	124		28	23	.094		.111	.105	97		32	95	6		5	3
D1	6.1	6.1	5.8		4.3	5.1	5.0		5.5	5.5	5.5		5.0	5.5	5.5		753	640	1777		240	208	286		.075	.056	.038		447	860	623		6	4	11	
D2	6.1	5.8	4.4		4.0	5.0	3.1		4.8	3.8	4.0		6.0	6.0	3.0		451	491	751		137	166	162		.105	.124	.094		269	289	-151		8	5	13	
D3	4.9	4.3	4.0		3.0	3.6	3.6		4.8	3.8	3.5		4.0	3.5	4.0		454	510	502		133	299	95		.108	.070	.092		-34	90	98		3	3	3	
D4	5.4	5.3	3.6		3.7	3.6	3.9		5.3	5.5	5.0		6.0	5.5	5.0		378	574	1006		137	204	98		.161	.062	.029		222	326	-706		8	15	2	

### Limitations

A technical limitation of this study was that subjects just played three of the four conditions. Moreover, the number of participants was restricted to sixteen subjects. All subjects were highly educated, young and healthy subjects. As this game is intended for rehabilitation training of stroke patients the results of this study cannot be directly transferred to a patient population.

Subjects reported that it was difficult to synchronize movements to the beat while playing the game. Movement-beat-synchronization has a potential to support motor training by keeping a stable tempo over a prolonged time [27]. Therefore, the haptics would need to be redesigned to enable an intuitive and easy synchronization of the movement to the beat. Different tempi choices may ease the movement-beat-synchronization.

The audio and haptic cues were designed to provide an equivalent conception of the task when visuals are not present. However, it remains unclear whether the introduction round in the C+V+ condition was confounding the results of the study.

We conducted the TTCT to receive a quantification of the participants’ creativity. The task in this study was specifically creating music. Therefore, the participants’ experience in making and creating music would have been of interest.

Another limiting aspect might be the restricted action space for arm movement. A more explorative behavior might have led to longer training times. However, the device is designed for motor training of subjects with moderate to severe arm weakness with a limited workspace. This might have lowered a feeling of movement freedom.

In our prototype game, we implemented music with a very simple rhythmic structure and mostly classical instruments. In the future, several music styles and genres such as rock, pop or classical music could be provided to meet subjective tastes and offer training variety.

## Conclusions

The combination of music and activities promoting creativity in motor training promotes enjoyment, and thus intrinsic motivation of subjects performing robot-assisted training. As the audio-haptic environment is sufficient to create a meaningful gameplay, music tasks can be performed without a visual display.

Promotion of creativity in a gamified task for neurorehabilitation may increase intrinsic motivation in patients but not training intensity in general. At the same time, omission of a visual display may not influence intrinsic motivation or training intensity. However, promotion of creativity differently influences training intensity dependent on the visual display of the task. When promoting creativity, audio-visuo-haptic environments lower training intensity while audio-haptic environments enhance training intensity.

We demonstrated the feasibility of playing an audio-haptic music game and suggest a follow-up study on stroke survivors.

## Appendix

### ARMin

The skeleton of the robot is designed according to the joints of the human arm, consisting of an upper arm link, lower arm link and a hand module. The upper arm link is connected to the fix robot body by a shoulder joint, and is connected to the other two links by an elbow and wrist joint. These joint-connections enable rotational movement of the exoskeleton links as 3D shoulder rotation, elbow flexion/extension, pro-/supination and wrist flexion/extension. The hand module additionally supports and assesses hand opening and closing. All seven degrees of freedom have two position sensors for redundant measuring of the current angle of the rotated link and actuators to move the links. The interaction between robot and the subject is transferred by cuffs at the upper arm, lower arm and hand module. The interaction forces are assessed by six degrees of freedom torque sensors mounted at the cuffs (Keller 2014). The ARMin robot can be adjusted to the upper arm, lower arm and hand properties. The same robot can be used in right arm and left arm configuration. The shoulder joint position can be set to the individual body size of the subjects. Safety features as mechanical endstops and software endstops for each joint, velocity limitations assure safe applications with healthy and impaired subjects. Using the position sensors and the actuators the ARMin robot features different controllers that support. ARMin is controlled with Simulink Realtime (Mathworks, R2014b).

### Summary of results

The detailed results are reported in Table 5.

### Sound examples

Soundalike 1 and 2 present an audio scene from the listening phase of a creation task: https://drive.google.com/open?id=0B3qqJ808s5veOVg2UzRCbFJaQVEhttps://drive.google.com/open?id=0B3qqJ808s5veQmVKS3pUY002Qjg
